# Informal ready-to-eat food vending governance in urban Nigeria: Formal and informal lenses guiding the practice

**DOI:** 10.1371/journal.pone.0288499

**Published:** 2023-07-13

**Authors:** Kehinde Paul Adeosun, Peter Oosterveer, Mary Greene

**Affiliations:** 1 Environmental Policy Group, Wageningen University and Research Centre, Wageningen, The Netherlands; 2 Department of Agricultural Economics, University of Nigeria, Nsukka, Nigeria; NUST: National University of Sciences and Technology, PAKISTAN

## Abstract

Informal ready-to-eat food vending is an important, cheap, convenient, accessible and readily available urban food supply sector that has become an increasingly important part of the diets of people in developing cities in Africa and throughout other contexts in the Global South. Over decades, despite challenges associated with health and hygiene, street foods have been informally accepted as part of the urban food supply system, particularly among the urban poor. Despite the importance of street foods to food security and employment needs in urban Nigeria and elsewhere, very little is known about the governance arrangements (whether formal or informal) revolving around their food provisioning practices. The paper explores governance arrangements that steer and shape food provisioning practices in Ibadan, Nigeria. Taking a social practice approach, the paper analyses the interconnections between governance and ready-to-eat food vending practices. It doing so, it draws on insights generated through a qualitative study incorporating in-depth interviews and participant observation methods to understand different governance arrangements revolving around food vending practices. The findings reveal that formal and informal governance structures are jointly steering and shaping practices of informal ready-to-eat food vending. They furthermore highlight the crucial role informal middlemen fulfill in informal food governance chains. These insights provide new avenues for thinking about food governance of urban food supply systems in terms of co-governance between formal and informal actors. They also provide empirical evidence that can aid policy application and implementation on urban food supply systems going forward. The paper concludes by discussing the potential of a co-governance informal food sector framework that recognizes and encompasses the formal-informal nature of the food sector. Such an approach recognizes and involves informal middlemen in the governance of informal ready-to-eat street food vending embedded in a larger framework of food system governance.

## 1. Introduction

The rapid growth of ready-to-eat food vending practices in the sub-Sahara African cities has been influenced by political economic transformations, which intersect with people’s changing lifestyles and the complex urban food system [1, 2]. Over decades, street foods have been accepted as part of the urban food supply system particularly among the urban poor, even though the sector is faced with challenges in terms of health and low diverse menu settings. The health status of ready-to-eat food vending is low because of provisioning low quality diets and few fruits and vegetables [3, 4]. Many street vendors are provisioning poor quality, unhealthy, higher-caloric, and low diverse foods [3, 5–7]. However, in many countries, street food vending (which is interchangeably referred to hereafter as ‘informal ready-to-eat food vending’ (IRFV) is yet to be included in the urban governance framework and, thus, as of yet, there is no policy supporting urban food vending practices [8]. The changing and increasingly complex intersecting socio-material dynamics of cities and changing urban lifestyles in Africa have positioned IRFV as an essential part of the urban food supply system. Particularly important drivers in out-of-home food consumption are low-income employment, rural to urban migration, lack of access to suitable housing for migrants, changes in political economic contexts and labor dynamics, including more women moving into formal jobs and increasingly flexible informal and precarious jobs among the urban poor, as well as challenging domestic and living conditions among urban poor consumers [1, 2, 9, 10]. IRFV has emerged in the context of these changing social dynamics and nowadays a large and growing number of low-income earner in cities in the global South depend on street foods [11, 12].

IRFV involves different categories of people selling self-prepared Nigerian meals, industrially-processed products like fruit juices, bread, and snacks, and unprocessed foods like fruits. IRFV practices operating outside the formal food provisioning system and mostly conducting business without legal status [13], have existed for decades in Nigerian cities. However, their presence was exacerbated during the structural adjustment policies in the 1980s when the contraction of formal employment forced many to resort to informal food markets [14, 15]. Informal food vending enterprises selling street foods are located throughout the urban landscape in Nigeria but are particularly concentrated in low income neighborhoods and can be found near busy places such as transportation hubs, office blocks, and school districts [16].

Food is overwhelmingly purchased rather than prepared by households. A large increase in patronage of street food has been recorded in Ibadan and other big cities in Nigeria [17]. This has been attributed to daily life practices, which involve long working hours and commuting, hence the preference for convenient, fast, easily accessible, and inexpensive food [18]. For instance, the city is encompassed by complex daily life activities due to the distance from the residential area to the workplace and inadequate housing facilities caused by overpopulation and rural-urban migration. Work and workplaces are mostly informally structured in low-income areas with no regular opening and closing times [2]. The urban poor are often locked into these complex daily lives with inadequate financial resources to overcome these challenges. This is also the situation in Ibadan city according to the study conducted by [2]. It has led to a high demand for vended foods in the Ibadan metropolis. Moreover, more people migrate to Ibadan on a daily basis without suitable housing infrastructure facilities. Many of them stay temporarily with families and friends but engage ready-to-eat food vending outlets for their daily meals.

Despite the growing importance of IRFV in urban life and the daily practices of consumers, the sector continues to be ignored by public policy. This is also true in Nigeria where the government is yet to see the importance of this sector as a channel to address food and nutrition security issues. Previous policies and programs on food nutrition and security in Nigeria have not recognized ready-to-eat food vending as an important part of the urban food supply system that influences the diets of the people [19, 20]. In the past, the government considered ready-to-eat food vending as sub-standard practices that should not be allowed due to certain shortcomings in terms of hygiene. This raised the level of persecutions by the government to restrict or disallow ready-to-eat food vending practices particularly in the city. More so, the available policy documents on food and nutrition security in Nigeria do not include any reference to the food vending sector among other food supply systems. In fact, the government is undecided on whether to formally recognize food vending or not. Despite the importance of informal food vending to food security and employment needs in urban Nigeria and elsewhere [19, 21–23], very little is known about the governance arrangements (whether formal or informal) that revolve around these food provisioning practices. Furthermore, it is unclear how these governance arrangements are structured and performed in practice as well as how formal and informal elements interrelate and co-exist in the informal food sector. In response to these knowledge gaps, this paper seeks to improve our understanding of IRFV governance arrangements [24]. Governance plays an important role in facilitating and supporting transformative practices and collective actions when they emerge. While there are studies on food governance and work on food practices, there is limited information on the interconnectedness between food-related governance and social practices, particularly in relation to street food vending practices.

Governance arrangements, including actors and tools, provide various support structures, for example through the provision of effective public policy strategies, coordination, and leadership [25] to everyday actions. The nature of governance arrangements around food practices differs depending on the category of the markets. For instance, formal markets are those that are regulated through formal governance structures where licensed sellers can publicly advertise their locations and prices, such as hypermarkets, supermarkets, and official restaurants [26, 27]. Formal governance is more-or-less structured with instruments of either state or local government (LG) to monitor and guide the activities of food vending actors. On the other hand, ready-to-eat food vending is a prevailing and distinctive component of the broad informal sector. Informal practices can be found in two key sectors, namely food retailing and ready-to-eat food vending. Informal food retailers are re-sellers, who often trade in raw food materials that they purchase either in formal or in informal markets and then process these inputs closer to the final consumer in order to increase their convenience [26–29]. According to [26], the informal food vending sector entails self-organized unlicensed food retail businesses. Informal governance include, in particular, involved vending association in regulating and guiding the activities of food vendors. Informal governance and components are organized by the vendors themselves without the influence of the government and they do not report their activities or receive instructions as regard the association’s operations from the government.

There is need for a better understanding of the informal governance arrangements in relation to the increasingly important IRFV sector. This paper situates itself as an initial empirical contribution to this gap in scientific knowledge. In doing so, the paper seeks to add insights into the contexts and practices of IRFV governance and how these enable and constrain informal ready-to-eat food vending practices. Thus, the paper aims to understand formal and informal governance arrangements and structures guiding and steering out-of-home ready-to-eat food provisioning practices among the poor consumers in urban settings.

While little work exists on informal food governance, a small body of work is emerging. The existing literature on food vending governance in Nigeria highlights the tensions and contradictions in the field, for example by revealing experiences of harassment among traders by local and state government officials, [14] whose efforts to exert control on the informal markets can lead to enforcement and the displacement of food vendors from government restricted zones. Existing literature on the governance of informal food vending tends to focus on laws and regulations [15, 23, 30, 31]. [15, 24, 32] these publications have looked at governance issues of food retailing and street food markets from government actors’ perspectives. [33] studied the governance of street food vending and the role of cooperation, the streamlining of vendor-authority relations and vendors’ livelihoods. Likewise, [34] studied the challenges and negotiating strategies of street-vended foods regulators and the implications for their relationship with street food venders. However, as of yet, there has been limited work looking at experiences and practices of governance as it interrelates with the everyday ready-to-eat food vending practices involving food vending practitioners and other stakeholders.

In seeking to explore the relationship between governance and street food vending the study adopts a social practices perspective (outlined in detail in the following sections), a framework that, despite some exceptions [7], has not been applied to the IRFV sector. Specifically, the paper seeks to understand governance dynamics in relation to social practices of selling street foods through empirical analysis in urban Nigeria.

Specifically, we explore existing practices of informal and formal regulations guiding the IRFV sector in the dynamic and growing city of Ibadan, with a particular view to understanding how these informal/formal governing practices intersect with and shape the daily practices of food vendors. Our analysis pays particular attention to the ways in which governance practices and IRFV influence the provision of good quality foods. In doing so, we respond to the call by [23, 35] for more nuanced approaches to capture the full spectrum of interactions between state actors and the informal food vending sector. Through applying a situated social practice theoretical perspective when investigating these dynamics, the paper advances this field both empirically and conceptually. Specifically, we aim to answer the following research question:

*In what ways do governance arrangements (formal or informal) steer and shape the out*-*of*-*home food provisioning practices of healthy and diverse ready-to-foods?*

The remainder of the paper is structured as follows: Section two explains the social practice theoretical framework informing our analysis of the interconnections between governance and ready-to-eat food vending. This is followed by a description of the study context and the methods used in collecting and analyzing the data. The final sections present and discuss the results of this investigation and, finally the conclusions and recommendations for policy and future research.

## 2. Theoretical perspective: intersections between governance and social practices

Taking a practice approach, the paper operationalized governance arrangements as governance practices. These practices consist of various (sub) governance activities and a series of actions that occur in time and space. These different (sub) governance activities and/or series of actions are referred to as elements of governance, which interact and integrate to form governance practices. Consequently, governance practices are considered to be composed of elements of governance, including rules, regulations, supervision, monitoring, control, and coordination, which are interconnected and coexist with everyday food vending practices as a bundle of practices. This paper analyses interconnections between governance practices and ready-to-eat food vending through the lens of social practice theory (SPT). Building on [36, 37], we progress a social practice approach that proposes that social structures such as rules and institutions do not simply ‘exist’ or influence actors ‘from the outside’, but are produced and reproduced in practice, in the interaction between actors and structures. In cities, government structures, policies, and regulations inter-play with everyday practices.

Applying a social practice approach allows us to better understand how governance practices shape everyday routinised actions rather than individual choices.

**Fig 1:**
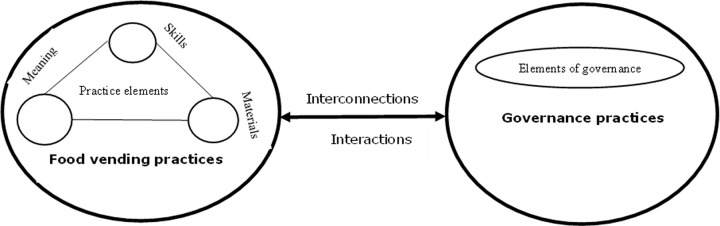
Dynamic interactions between governance and food vending practices.

Through this SPT lens, we explore how governance interacts with specific components of social practices. Practices are viewed as comprised of distinct set of elements (meanings, competencies/skills and materials) that interact and combine in performances of actions. Following this, we explore how governance practices and/or elements of governance interacts and interconnect with food vending practices and/or elements of food vending practices and a series of actions within food vending landscape arranged as a bundles of practices, see Fig 1. For instance, rules and regulations are instructions, pathways, and principles that shape how food vending practices are performed, thereby guiding, directing, enabling, and constraining individuals involved in these practices [38]. This study also elaborates on how elements of governance interact and interrelate with elements of food vending for the desired outcome(s) (see Fig 1). Governance highlights how agency and power relations among different actors are key to any transformative change in food vending systems. The outcome of a practice is driven by the effectiveness of rules that govern it. Hence, a well refined outcome can be achieved if there are effective and robust interactions between the practices, sub-practices and the governance.

The shift from government to governance is by now well documented [39–42], with both market actors and civil society organizations taking on new responsibilities in the governance of societies and organizations, next to government actors [43]. Street food vending governance therefore also informs efforts to shape, improve or sustain (the reproduction of) ready-to-eat food vending practices in terms of quality of the diets provisioned. Actors involved in these practices reproduce them by drawing upon specific sets of rules and resources constitutive of these practices [44].

Consequently, the study explores how rules, regulations, supervision, monitoring, and coordination are embedded in governance practices and work to co-shape IRFV practices in terms of their daily operation, functioning, and performance [45]. Specifically, the paper seeks to explore how different critical elements in everyday food vending practices such as food outlet cleaning, raw food material procurement, food preparation, and menu setting are governed and shaped by sets of governance practices on a daily basis.

## 3. Methods

### 3.1 Study location

This investigation was carried out among low-income urban residents in Ibadan, a dynamic Nigerian city with important social and economic functions. It builds on previous studies on the social dynamics of food provisioning in terms of experiences, performances, and functioning of food vending in Ibadan [2, 7]. Ibadan, a city experiencing rapid growth through a process of peri-urbanization, has a population of about 4 million [46]. The city is highly multicultural, bringing together people from different cultures in Nigeria and beyond. Ibadan is home to different research institutes, including the International Institute of Tropical Agriculture (IITA). Thus, Ibadan is well-positioned to be globally competitive and ‘comparable to other major cities in the world’ [47].

Administratively, Ibadan consists of eleven Local Government Areas (LGAs), seven located in the Northern part of the city, three in the Southern part, and one at the boundary between the Northern and Southern parts. The study selected poor urban communities because previous research (as outlined in the Introduction) has revealed that ready-to-eat food vending is the most prominently used food provisioning outlet among low income consumers. Most people in the communities selected live below the global urban poverty line of $1.90 per day [48]. They also predominantly live below the Nigerian national minimum wage of Naira 30,000 ($77) per month and far below the Nigerian average income of Naira 339,000 ($880) per month [49, 50]. The administrative and commercial functions of Ibadan transcend state boundaries and have important social and economic functions.

### 3.2 Sampling procedure

A preliminary survey was first conducted before the main data collection to map and understand and familiarize with the study location. Community leaders were consulted to inform them about our presence and purpose. They were provided with an information sheet detailing the purpose and expected outcomes of the research, and how their involvement would facilitate its success. To have a good representation of the study area, the study selected two LGAs from the Northern part of Ibadan where seven LGAs are located, one LGA from the Southern part of Ibadan where three LGAs are located, and one LGA from Central Ibadan, located at the boundary between the Northern and Southern parts of the city in the sampling frame. Thus, in total, four LGAs were selected as the sample area for the study and within each of them, two poor urban communities were selected as sites for the study to make total of eight communities. We mapped the food vending outlets in these selected communities by recording and illustrating their coordinate locations and geographic spread with the aid of Geographical Information System (GIS) software. A total of 686 food vending outlets were mapped. These were categorized according to vending type and included 319 traditional food vending outlets, 268 processed food vending outlets and 98 unprocessed food vending outlets. The purpose of this initial mapping stage was to visualize the food vending environment in the selected communities and to aid the sampling of food vending outlets for participating in the survey and qualitative aspects of the study.

**Fig 2:**
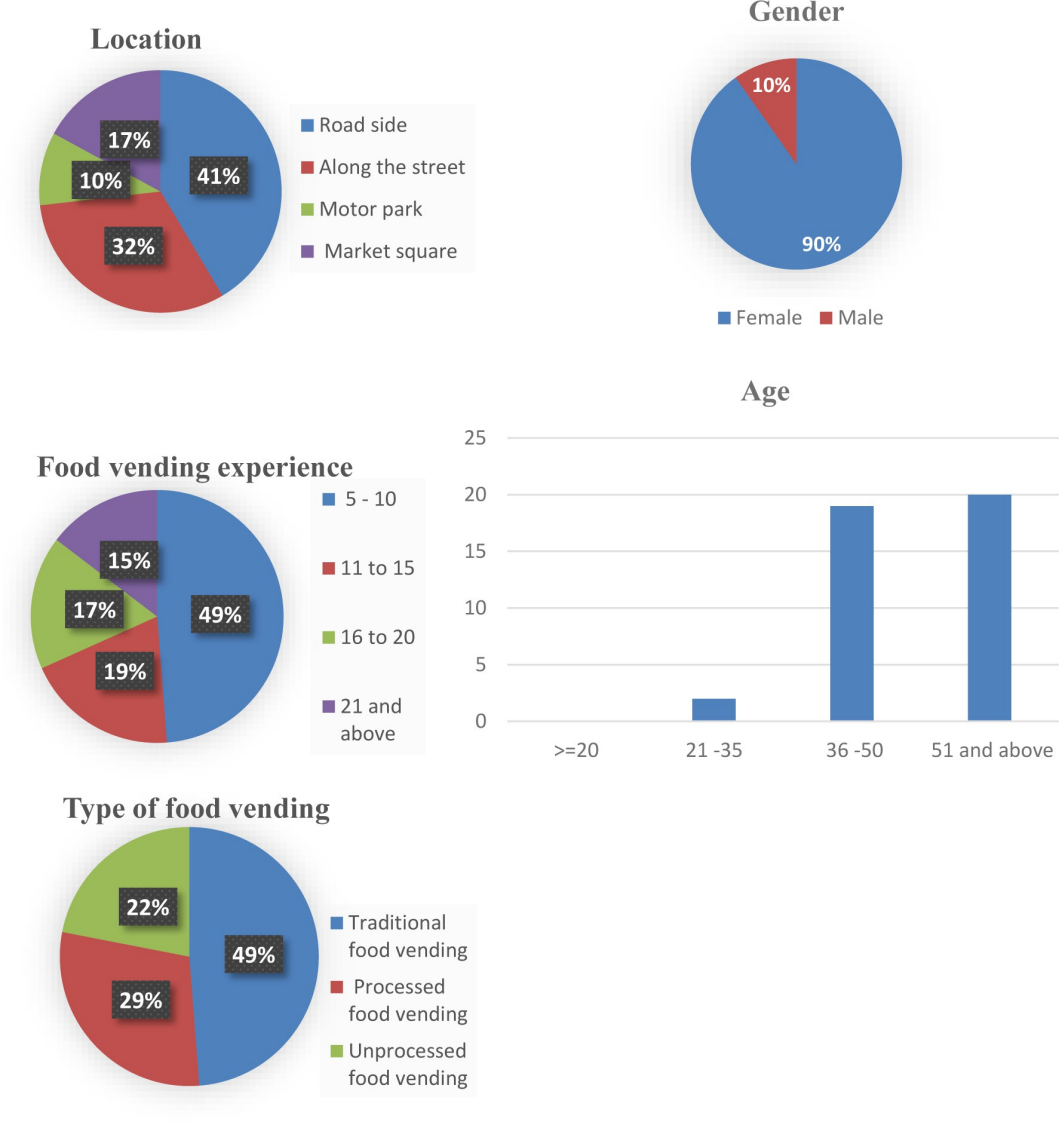
Socio-economic characteristic of food vendors in Ibadan.

Following the GIS mapping, a socio-economic survey of the vendors was conducted. This survey enabled us to further map the vendors according to key socioeconomic indicators include age and gender of the vendor, food vending experience, location of food vending outlet and type of food vending category (traditional, processed, unprocessed) (see Fig 2). These insights were used to identify participants for the qualitative aspect of the study (see below) and harness a wide range of experiences and perspectives about governance issues at different socio-economic levels of food vending that affect food vending practices in the study area. The informal food vendors included in this study are individuals directly in control of food vending outlets and in operation for at least five years to ensure sufficient information dynamics and up-to-date experiences.

#### 3.2.1 Socio-economic indicators of food vendors

Regarding socioeconomic characteristics of the selected food vendors, the majority (90%) of the food vendors surveyed were female. This is in line with findings of previous studies that have reported high levels of women’s involvement in street food vending [7, 12]. Likewise, in terms of spatial location of food vending activities, most food vendors are concentrated along roadsides (41%), along the community streets (32%), and in market squares (17%), and motor parks (10%). Regarding the experience in food vending, 49% of respondents have been working in the food vending sector for 5 to 10 years, while 19% have been in food vending for 16 to 20 years. The results of the overview of the socio-economic indicators are presented in Fig 2.

Based on this survey data, forty-five (45) respondents were purposively selected for further qualitative research involving interviews and observations to explore practitioners’ practices and experiences in-depth “see Table 1”. Specifically, forty-one (41) food vendors were purposively selected across the different categories of food vending and socio-economic indicators and five of them are among the food vending association leaders. The categories of food vendors included 19 traditional meal vendors, 12 processed food vendors, and 5 unprocessed food vendors (See [7] for an overview of the different categories of vendor). Likewise, three (3) LG agents and one (1) state actor were selected for expert interviews (see Table 1 for an overview of respondents selected for the study). These are actors that have links with food vending activities in the urban area.

**Table 1:** Overview of practitioners and stakeholders interviewed in this study.

Respondents interviewed	Number
Food vendors	36
Food vending association leaders	5
Local government agents	3
State agent	1
**Total**	**45**

### 3.3 Data collection

The insights discussed in this paper are based on the qualitative data generated through the semi-structured in-depth interviews conducted with vendors and expert interviewees (outlined above) and passive participant observation. Observations were conducted at the selected food vending outlets providing information on the situational dynamics that the interviews may have missed. Interviews with respondents were structured to generate insights into IRFV governance. Across the vendors, association leaders and government agent’s interviews, the study explored interviewee’s perspectives and experiences with regard to rules and regulations governing the food vending sector and how this plays out in daily practices and operations.

The wider study from which the data in this paper is drawn has been approved by the Wageningen School of Social Science, with all ethical considerations addressed, including obtaining informed consent from all participants, ensuring participant anonymity and implementing secure data management practices. Given that it was a survey and not a human experiment and because we obtained the respondents’ consent, within the Wageningen University ethics procedure, the study did not require further approval from Wageningen University’s Human Experimentation Ethics Committee (HEEC).

A practice-informed interview schedule structured the discussions on governance practices and their interrelations with ready-to-eat food vending practices. Each interview lasted approximately 30 to 45 minutes. Interviews with vendors and association leaders were performed at their food vending outlets, while, the state and LG actors’ interviews were conducted at their secretariat’s offices. Data was collected in the period from June to December 2021. Although, this study was conducted during the COVID-19 pandemic, during the period of interviewing, all lockdown measures had been officially lifted in Nigeria and the food vendors were operating as normal. In addition, all precautionary COVID-19 measures were taken with the interviewees, including maintaining1.5 meters social distancing and the wearing of facemasks by respondents and interviewers.

The interviews were recorded, transcribed and content analysis with inductive coding was used to code the transcribed interview data using qualitative analysis software (Atlas ti.). The open codes that were identified were then analyzed, compared, and grouped into categories. An iteration between inductive and deductive analysis was used for particular themes and patterns and then reviewed to further categorize this data according to the SPT framework.

## 4. Results

The results from this study reveal insights into the practices and experiences of the food vendors, food vending association leaders, LG actors and state ministries. In particular, they explain how formal and informal governance interacts in co-steering the food vending practices in Ibadan, Nigeria. Formal regulations are government-stipulated rules that revolve around informal food vending practices. Likewise, informal rules are mostly initiated by food vendors themselves, particularly through their association. Altogether these rules shape the everyday practices of food vending in the city. There are two food vending associations in Ibadan: one that includes all the traditional and processed food vendors and another that includes all unprocessed food vendors, but both have similar modus operandi and rules and regulations guiding vendors activities.

### 4.1 Rules’ enactment and implementation practices: Formal and informal lenses

The formal coordination, monitoring, control and supervision of food vending activities reside within the state ministries and LG institutions. The local governments and state ministries operate rather independently, but the LG is more involved in the regular coordination and supervision of food vending activities. While both actors visit and monitor food vendors, LGs engage in this more frequently on a weekly basis.

“……*Both of the arms of government officials visit me (i.e. Public Health officials or Environmental Sanitarian)*. *But the local government officers visit me regularly, especially every Thursday of the week (food vendor 3, traditional, experience 10, female)”*.

Despite food vending being an informal sector, there are different arms of the government involved in the supervision and coordination of food vending activities. The LG agents report that they are interested in this particular sector because of the kind of services that sector offers. The government agents highlight that the food vending sector feeds a large number of people but the consumers don’t have control over the foods they get, in terms of how it is being prepared, the cleanliness of the water used, and the hygiene of the food environment. More so, any contamination coming from this sector negatively impacts a large number of people. Hence, they stress that regulating the sector, through public health officers who are in charge of environmental cleaning and personal hygiene, and task force officers who are in charge of collecting levies, is critical. However, this process is sometimes contentious and tensions between government actors and vendors have occurred. Conflicts between government officials and vendors occur because sometimes the agents are forceful or hard on the food vendors and the food vendors consider some of the activities of the government agents as oppression. Due to several confrontations and altercations between the LG and vendors a decision was made to how the levies are paid. Instead of LGs collecting levies from individual vendors, the levies are now paid via the food vending associations to the government. The associations have their mechanisms of dealing with their members without rancor.

Even though the rules are not handily available to the food vendors, government officials continue to enforce regulations through verbatim means. The government officials confirmed that the rules guiding food vending practice are written with other food sectors’ rules. However, these documents are not available to the food vendors. This implies that there is as yet no standardized regulatory framework specifically for informal food vending activities. This was confirmed by LG officials who indicated that only official restaurants are licensed, standardized and registered with them. Nevertheless, despite the informal character of the food vending sector, government officials still seek to enforce and regulate it. Monitoring and supervision are enacted through enforcing “rules” that seek to regulate the quality of the food prepared, the cleanliness of the food vending outlet, the health status of the food vendors to be checked in government-accredited hospitals, and proper waste disposal to ensure the safety of the people living in the community where the canteen is located. These rules also emphasize how the food vendor’s environment must look and how food vending workers should be dressed. Vendors indicated that LG officials visit their food vending outlets every Thursday of the week, to check if they are abiding by the rules. Daily, regulators face challenges that limit the implementation and effectiveness of the regulations. For instance, officers reported that they don’t have enough staff to do the supervision. This is in line with [34, 51] who found that regulators operate in a context with limited resources, leading to a general feeling of neglect and inability to implement food safety regulations.

Even though rules and regulations concerning food vending activities are made and enforced by the government, to ensure more effective governance this is done in consultation with the food vendors, particularly with the food vending associations’ leaders. The leaders of the food vending associations meet with the government when the need arises to deliberate on different regulations that are applied to them, on the implementation procedures or on any other important issue. The importance of inclusive food governance was also revealed in the findings of [52] that retailers and local authorities’ interest in having to demonstrate their mutual role in supporting local producers. A vendor reported that anytime the government wants to make a new rule, they will gather all the food vendor associations’ leaders to discuss this with them. Association leaders have a crucial intermediatory role; whatever the association leaders discuss and conclude with the government is passed down to the rest of the food vendors during association meetings. According to [53, 54] a street vending association can be characterized as one of negotiation, where street vendors and city regulators are constantly negotiating over space for business, economic advantages, and power.

“…….*The government officials usually invite us for discussion and opinions on matters that border on customers*’ *welfare and food vending best practices and we come back to give feedback to our members in the association meetings to inform them about the new rules (association leader 2, traditional-processed, experience 20, female)*.

The vendor respondents predominantly indicated that they have confidence in their association leaders’ ability to represent and defend their interests. On the other hand, food vendors reported the challenge they face in terms of high prices of food raw materials, as almost all food vendors complain that the price of food materials is increasing every day and negatively impacting their business.

#### 4.1.1 Hygiene practices in the food vending outlet

As stipulated by the government rules guiding the sector, food vendors are required to keep their environment clean. Interviews with vendors also confirmed by government officials indicated that they must make sure they sweep and dispose of their waste regularly and do not pile up any waste around their food vending outlet. This is enforced by government officials who visit the vending outlets and inspect whether the vending outlet environment is sufficiently clean and well maintained. This also applies to their practice when selling foods; vendors indicated that officials instruct them on their physical hygiene and cleanliness: their clothes must not be dirty, they must cover every part of their body and their nails must not be overgrown. Likewise, they must not converse when serving, carrying, cooking, and dishing out foods to the customers to avoid spitting saliva on the foods.

“……*Yes, there are existing rules on dressing and the other physical appearance of the food vending staff*, *how healthy they must look or appear, and avoiding involving sickly staff members in the daily activities of the business until they are well again”, (public health officer, female)*.

“……*I was instructed by the government officials anytime they visit on how to maintain my food outlet*, *and that my food outlet should be clean always”, (food vendor 1, traditional, experience 11, female)*.

Observations at the food vending outlets revealed that most vendors strictly observe these rules; the food vending outlets were clean, they attend to customers and to the siting arrangements. This confirms that the food vendors are aware of these rules and execute them, and aligns with the findings of [55] that food vendors are aware of good basic hygiene practices despite having low levels of literacy and income, and limited job security.

#### 4.1.2 Food preparation and menu-settings

The respondents reported that all vending staff involved in food preparation and using equipment for food processing and preparation must practice good hygiene. They must adhere to food safety and not use rotten raw food materials, while unprocessed food vendors should avoid selling spoiled fruits and using harmful substances like methane to ripen or preserve their fruits [56]. the quality of raw materials used in the preparation of street foods is very important to food safety as contaminants can persist even through preparation and cooking.

“……*The rules by the government that guide our food vending practices here are basically on the provisioning of healthy foods*, *personal hygiene of all members of staff, and general cleanliness of the canteen environment, (food vendor 7, processed, experience 12, female)”*.

At the time of the interviews, there were also new rules due to the evolution of the Covid19 pandemic. The canteen must not be overcrowded, vending staff must regularly wash their hands with soap under running water and vendors have to check customers’ temperature with an infrared thermometer. The customer sitting arrangements also had to be modified to enable 1.5m distance between customer groups.

### 4.2 Interrelations between food vendors and government officials

The findings indicate that there is generally quite a cordial relationship between food vendors and government agencies, with food vendors generally obeying government-stipulated rules and regulations. However, there are still challenges faced within this relationship, particularly, as outlined above, in the area of levy collection and issuing fines. Food vendors always face serious opposition by the LG if they don’t pay their levy when due. Vendors view these fines as being far too heavy for a late fee. Despite this ongoing issue, there is a cordial relationship between the government and the food vending associations’ leaders as they help the government to monitor the level of compliance to government-imposed rules on the food vending practices and collect some levies on behalf of the government. This aligns with [33] who identified a non-confrontational relationship between some groups of vendors and the Chengguan city authority. Rather than one of conflict and opposition, their relationship is better understood as one of cooperation. Similarly, [15] found that informal food vendors in the smaller urban centers in Nigeria operate in an enabling environment for their activities with less harassment by government agencies compared to a large city like Lagos. Harassment mostly happens to vendors who want to sell in government restricted areas or along busy roads.

“……*Basically, the association ensures that every member complies with the government rules, and any grievance among members should be reported to the leaders for settlement*. *We have a task force that moves around to ensure compliance among members, (association leader 2, traditional-processed, experience 20, female)”*.

In addition, food vendors collaborate with other actors in the private sector, particularly with microfinance institutions to get a loan to support their business against a low interest rate. Even though interest rates vary over time, they are always lower than the rates of commercial banks. Likewise, the repayment pattern is small-scale business friendly. There are other companies like a bakery, a pure water factory and Coca-Cola that are interested in collaboration on business grounds. They connect to food vendors basically because they expect food vendors to buy their products to complement the ready-to-eat foods they provisioned.

“… ..*their interest is basically on the patronage they expect from me to buy their products to complement my foods*. *The nature of their interest is basically on business matters*, *(food vendor 15, processed, experience 9, female)”*.

For instance, some companies collaborate with food vendors by supplying whole food ingredients and related information on ingredient measurements to ensure health and safety to their customers.

### 4.3 Self-organization in food vending practices

Informal coordination, supervision, control and monitoring of food vending mostly resides with the food vending associations. Everyone selling ready-to-eat foods must belong to an association to avoid trouble, including having their outlet and food seized by the association. The leaders of the associations have data of all food vendors, therefore, coordinating them is not a challenge. They have their way of detecting those who have not registered as a member. For instance, whenever members notice an unfamiliar face selling food, they approach that individual to register with the association to avoid being stopped by the association task force. In case he/she refuses, the member will report this to the association leaders who will take action and request the association task force to stop such person from selling. The two existing associations have key executive members including the president, vice-president, treasurer, secretary, and taskforce officer, who direct their day-to-day affairs. The association leaders are food vendors themselves and they have been involved in food vending practices already for a long time. They have ascended into their position based on their years of food vending experience. As observed, most members of the executives are elderly. The young food vendors see the elderly ones as matrons and they follow their guidance. During the associations’ weekly meetings, individual food vendors with ideas on how to improve their common practices can share these to help others.

“……*Yes, am a registered member, I have my certificate and I always pay my levy to the association*. *Yes, I do attend meetings every Thursday of the week. The association comprises of traditional and processed food vendors and always sees to the affairs of the members, (food vendor 4, traditional, experience 6, female)*.

Apart from government rules, associations also have rules that guide food vending practices, although some of them align with the government rules. Members selling food close to one another must be at a distance of at least five to six poles (about 40 meters). All members must fully participate in all activities and meetings of the association and are not allowed to arrive late or be absent from the weekly meetings without prior notice.

“……*association rules apart from the ones that border on members punctuality and some few social activities in the meetings are aligned with governments rules*, *(association leader 3, unprocessed, experience 22, female)*”.

The associations monitor the types of food provisioned to ensure compliance with the stipulated norms among the food vendors in the same location in order to maintain peaceful coexistence between them. The associations also provide a platform of support for members who lack survival or coping strategies when facing challenges in their business. In turn, members of the associations who have in the past successfully overcome business challenges can volunteer to help others. According to [57] associations can help their members develop their capacity, analyze complex issues, and provide access to information about important community or public issues impacting their livelihood. The experience of the vendors studied in this research resonates with this.

On the other hand, some food vendors report that they are not comfortable with the activities of the associations, particularly regarding compulsory attendance to meetings and the mandatory weekly contribution. There is hardly any information about the purpose the association is using the money for. While not as prevalent as positive experiences, the negative experience of these vendors resonates with insights reported by [57] whose research in Lima found that street vending associations can suffer from low levels of participation, high rates of exiting members and a lack of trust between leaders and members.

“……*The challenge I used to face from the association leader is the compulsory attendance at the association meeting*, *(food vendor 16, unprocessed, experience 5, female)*.

#### 4.3.1 Association’s recruitment practices

Before starting food vending, a vendor has to pass through the entrance processes in the association. The association takes the vendors through (paid) training and seminars to ensure they become familiar with their operations. During this process, the vender is introduced to the other members and a certificate will be issued. The vendor registers with the association by paying a one-time fee of #10,000 (24 dollars), two cartons of biscuits, and a crate of soft drinks. The items provisioned are similar for each association but the “size/quantity” may vary between them. The vendor will also be provided with an association membership card and a printed sheet outlining the association rules. From that moment on, the person has made a commitment to abide by these rules that guide the association and the food vending practices.

#### 4.3.2 Training on best food practices

Periodical training in the form of seminars or workshops on different aspects of food vending are organized by either the government agencies or food vending associations. Environmental officers organize training for food vendors on the best practices in food vending outlets. This training is offered once or twice a year and covers topics such as maintaining the cleanliness of the food vending environment, personal hygiene, dress code, provisioning of quality food, recipes for preparing stews or soup in healthy and nutritious ways, and how to effectively relate with customers. To ensure safe food for the consumers, health education of the vendors is needed [6].

“……*Yes, I remembered they also trained us on types of ingredients to use for the health benefits of the consumers*. *Quality ingredients used in appropriate quantity will result in healthy food for public consumption, (food vendor 2, processed, experience 7, female)*.

Food vendors reported that they usually do the training in LG offices close to their vending locations. Many vendors felt that the application of knowledge from this training has improved their practice and supported them to develop good customer relations. More so, the training serves as an avenue to motivate them to do more and aim for best practices. Vendors usually pay for the training which provides access to training materials and a certificate, which is given after completion of the training. The certificate is valid until another training is being organized and enables the monitoring officials (task force) to identify those who have attended the training and those who have not. There is a penalty for vendors who refuse to attend the training and they are not allowed to practice vending until the next training.

“……*I do attend the training not just to avoid being sanctioned but to learn lessons that can help me attract customers*, *as they will only patronize food vendors whose environment is appealing and provide tasty foods*, *(food vendor 5*, *traditional*, *experience 11*, *female)”*.

The government usually organizes the training in consultation with food vending association leaders so they can help to organize it and mobilize their members. They provide the associations’ leaders with the agenda for the training and the amount to be paid by each food vendor to attend the training. During the training, selected operators from a more conventional restaurant are contracted to train them. These standardized, licensed and registered restaurants. though expensive and rare in the area, are found in the cosmopolitan area (core-center of the city where the rich people reside). Likewise, the association sometimes organizes food vendors and prepares them to be addressed by a food-related Non-Governmental Organization. These activities contribute to “cohabitation”, though which informal food vendors coexist with, and benefit from formal food vending activities, such as supermarkets and restaurants, which strengthens their resilience [58].

“……*A big restaurant like Iya Dunni is to provide us with training for a smaller food canteen like me*. *It serves as an avenue to encourage and motivate me to do more and aim for the best practices, (food vendor 10, traditional, experience 6, female)*”.

The COVID19 pandemic has brought some changes in their food vending practices, so they likewise receive education on how to cope with the new guidelines, particularly with respect to the sitting arrangements in the vending outlets and the hygiene practices within the food vending facilities.

### 4.4 Levy and defaulting

A small amount is being paid as levy (average #300 (0.70 dollars per day) by the food vendors to the government. However, the food vendors on average earn in the range from #1,000 to #2,500 (2.5 to 6 dollars) per day. As already outlined, in the past this levy was paid directly by vendors to the government agencies. However, due to clashes between food vendors and government agents, this process now occurs through the food vending associations.

“……*Before the food vendors association was saddled with this responsibility of collecting money on behalf of government officials*, *the government agencies used to come directly to approach us [the vendors] to pay local and state governments levies but* …*due to clashes between food vendors and some of these impatient government officials it was later decided that the association should take up this responsibility, (association leader 4, traditional-processed, experience 19, female)*”.

This type of tension between formal actors and vendors in an informal sector has also been reported in other food vending contexts. For example [59]’s study in Guangzhou, China revealed that a stringent regulatory environment and enforcement of levy payments by government officials was met with vendors’ resistance, which sometimes escalated into violent clashes.

“……*the only challenge that I am facing from the government officials is the payment of levy because*, *how much am I realizing from what I am selling and they will still come to collect money, (food vendor 21, traditional, experience 17, female)*”.

In Ibadan, to reduce conflict, the vendors associations reached an agreement with the government on the modality and the amount of the payment. The associations’ leaders levy their members on a zone by zone basis, with options to pay monthly or annually, and issue them receipts upon payment. Upon the collection of the payments, association leaders pass the money to the LG for onward transfer to the state government account after the LG has taken their share.

The levy money constitutes a form of revenue generation from vending sector to the government. The specific amount paid, differs according to business type and size. The flat rate figure that is expected to be paid by individual vendors is determined by the local government in consultation with food vending association leaders. The government sees this payment as a simple civic responsibility of citizens.

“……*I pay levies to both state and local [government] on monthly basis through our association*… *I pay #200 to the state government purse and #100 to the local government purse both on monthly basis, (food vendor 13, traditional, experience 9, female)*.

Even though they directly or indirectly pay some sort of levy to the government, food vendors still operate as an informal sector. They don’t pay tax and remain unlicensed, unstandardized and not officially registered with government. They don’t submit a report on their activities to the government, so they are not accountable to the government. For these reasons, despite efforts to reduce conflict by mediating the payment via the association, many vendors still question the government levy payment with many reporting that given their status as low-income earners the levy is too high. They question why they should pay anything to the government when they remain unregistered and do not benefit enough in terms of financial and/or infrastructural support.

Penalties are issued in cases where there is a default of the levy payment or when an environmental or hygiene regulation is broken. Penalties and other stick measures are issued either from a government agency or the vending association task force. The association task force monitors the level of compliance among association members and checks whoever is yet to pay his/her levy and reasons for instances of lateness before a proper punishment is given. Beyond late levy payments, there are punishments for defaulting existing rules (for example in relation to violating healthy, safety or cleanliness regulations) laid down by the government and the association. When vendors default, the association task force or government agencies usually confiscate their food and seal their shop so they cannot continue selling until they go to the LG office and pay the required fine.

“……*Like some time ago they locked my neighbor*’*s shop because she put small dirt inside the sachet water nylon pack in front of her shop and they ask her to pay #1000, (food vendor 9, traditional, experience 13, female)*.

Apart from the levy to the government, food vendors are also required to financially contribute to their association through associations’ or members’ support. This money is used to pay for activities performed for the collective by the association, for example, covering clothing and transport fares for association representatives to attend meetings with the government. Likewise, if any member of the association needs urgent support or faces a fiscal emergency with their business, the members’ collectively support them through the association contribution.

“……*We pay #200 weekly to the association. This money is used to buy clothes for our social functions*. *Also if anyone needs assistance, we give the person support from the money contributed (food vendor 14, unprocessed, experience 8, female)*.

### 4.5 Self-directed practices in food vending outlets

The stipulated rules and regulations by the government and food vending associations further stimulate individual food vendors to include, extend and expand on other practices that can add value to their food provisioning activity. They are extending the required practice activities beyond initial instructions and their implementation is solely based on the discretion of individual food vendors. These practices are undertaken to further increase customers’ patronage as well as acceptance of out-of-home food provisioning. They cut across but are not limited to the type of food items they provision and respondents report that they make rules for their staff based on hygiene and punctuality. Some of the respondents do not allow customers to remain in their food vending outlet after eating to avoid customers crowding at the outlet. They also monitor their staff on their cooking practices and handing out menus to the customers to maintain hygiene standards. Whenever customers buy at a food vending outlet, he/she cannot return the food because it is likely to have been contaminated, so it is unacceptable to return the food bought. This is further linked to the health and food safety practices of food vending.

“… ..*My own self rules that am following is when I employ workers, I tell them that they must not leave the plates the customer used to eat without washing them with immediate effect*. *I also instruct them on the number of ingredients to be used for soup and other things, (food vendor 18, traditional, experience 14, female)*.

Analysis also revealed that societal norms guiding appropriate food vending practices with a particular community also shape how vendors operates. For instance, some communities have a specific time at which movement is not allowed for security purposes or prohibit vendors selling late at night along community and neighbourhood streets. These informal norms and rules are communicated and monitored by community leaders.

“… ..*Yes, there are community norms. The norm is that I must not stay here till late in the night*. *They only allow me to stay from 6 am to 8 pm. I usually ensure that I abide by the time stipulated by the community leaders, (food vendor 17, unprocessed, experience 7, male)*”.

There is also a norm that the food vendors should prevent rivalry or any kind of unhealthy competition among themselves. Vendors keep to the rules guiding the community where they undertake their practices.

## 5. Discussion and conclusion

This paper aimed to gain an in-depth understanding of the (formal and informal) governance arrangements revolving around food provisioning practices in Ibadan, Nigeria. Likewise, the findings of this paper demonstrate that governance and social practices can interact and co-exist together to shape a desired outcome. Exploring the perspectives and experiences of practitioners and stakeholders in the food vending sector, the findings address gaps in understanding concerning how the governance of the increasingly significant informal food sector plays out. In addressing gaps in research, the insights above illustrate how formal and informal governance elements intersect through practices that oversee the coordination, supervision, control and monitoring of food vending activities. Where there are concerns about out-of-home food provisioning practices in terms of their health and safety, the government and food vending associations seek to steer and direct food vending practices to improve them and make them more acceptable. Despite this form of co-governance between formal government and the informal association, some food vendors are not comfortable with some of the rules and regulations guiding their practice, particularly on the levy payment and how fines are been issued. They consider themselves as being informal who, unlike more formal food businesses, receive little or no benefits from the government in terms of support for their business. Despite paying money to the government, they feel they contribute to remain mostly being neglected as a sector. The food vendors reported that they would prefer that the governance of their sector remained fully within their association who they see as having more appropriate informal mechanisms of dealing with issues, including on how to guide and govern their practices and assist with the implementation of rules and the resolution of disputes. Decentering governance to the associations would likely limit the tensions generated between government agents and food vendors and prevent further confrontations.

Despite the informal nature of the sector, formal regulations are established and enforced by the government to guide food vending practices. These regulations, which govern the everyday practices of food vendors, are brought to the notice of food vendors by government officials who monitor if they are being implemented and followed. All food vendors are expected to obey the rules to avoid penalties. These formal governance dynamics within an informal food sector align with the findings of others. For example, [60] reported in a study conducted in South Africa that the formal governance of street foods lies with Public Health officers as well as the Food and Drugs agency in LG who have the power to confiscate any foods that are deemed unsafe and unfit for consumption, and to fine traders accordingly. Even though the status of food vending is still informal in urban Ibadan, the government interferes with individual food vendors’ activities and controls them directly and indirectly through the associations. The government maintains the power to stop any food vendor that goes against the rules and regulations and can sanction individual food vendors. The government agents are driven by an understanding that, when implemented, the government rules and regulations can bring about improved health and food safety practices in the informal food vending sector. Indeed, the analysis suggests that the active supervision and monitoring of ready-to-eat food vending has re-shaped and influenced food vending practices in terms of norms of cleanliness, hygiene, promoting clean food outlet environments, and maintaining hygiene in food preparation practices. This highlights that formal and informal rules and regulations can co-shape the norms and logics guiding everyday actions promoting effective, sustainable, and continuous practices [45].

The study analyzed the different formal governance arrangements that co-exist with food vending practices in the city of Ibadan. The analysis revealed that the city authority is aware of the operations of food vending and directly and indirectly oversees their activities, particularly through the government-stipulated rules and regulations that guide food vending practices. The government’s interest is focused on the informal food sector because of the nature of the services it provides and the impact the sector is already having on the larger society, particularly for low income out-of-home consumers. Due to the rapid rise in this sector, any contamination coming from informal food vending can negatively impact the larger society. Consumers only have access to the final product and cannot change the product before consumption compared to food retailers who resell raw food materials to households who can prepare the final product themselves. This suggests that if this sector is not properly monitored, it can spread food poisoning in the community with huge costs to the government. This implies that the intersections and interconnections between governance and practices could aim to shape a refined outcome. Hence, governance plays a crucial role in steering practices for the effective delivery of their outcomes. On the other hand, another important element of food security was missing in the overall ready-to-eat food vending governance arrangements. The food vending diversity provisioned has received little attention from the government; although the association confirmed they do talk about food diversity, it is not the first on their priority list and much depends on the discretion of individual food vendors. Food vending stakeholders revealed that they have not taken cognizance of aspects of food diversity in their out-of-home food provisioning practices. However, previous studies by [2, 7] have suggested that food vending diversity is important for ensuring balanced diets and diverse nutrient intake by out-of-home food consumers. This becomes even more important as more people in urban areas consume their meals out-of-home daily.

The findings in this paper suggest that formal rules constrain and influence food vendors to stick to acceptable practices, even if they want to do otherwise. The government-stipulated rules and regulations streamline food vending practices to more acceptable variants. The majority of food vendors expressed that they are aware of these rules and want to follow them to continue their practice and to avoid being fined by the government or the association task force. More so, when followed the required rules will attract more customers to their food vending outlet as it is likely to increase their trust in out-of-home food provisioning in terms of food safety.

The study revealed that the governance of food vending in urban Nigeria is a joint action involving formal government officers and informal food vending associations. Most government decisions about food vending are taken in consultation with the food vending associations. This depicts that for formal rules to work effectively for informal food vending activities, informal stakeholders should be incorporated in the decision-making process. [23] there are few instances of informal vendors forcing change in formal legislative and regulatory frameworks governing the informal food sector, including how or whether they are implemented. Thus, the effective implementation and sustenance of the rules and regulations concerning informal food vending practices lie within the ambit of both the government and informal food vending associations with the latter playing a crucial role. This resonates well with the findings of a study conducted in China by [24] who found that the public-private hybrid model of urban food system governance is highly inclusive as it involves numerous food vendors in the system of urban food provisioning. The results from the study suggest that involving informal actors in the enactment, execution, and implementation of the rules and regulations will propel their quick adoption. This indicates that interconnections between the (formal-informal) co-governance and food vending practices are indispensable to achieving acceptable and effective informal food vending systems. This study provides empirical information on how the governance process of informal food vending can go forward. This is similar to the findings of a study conducted in Nanjing, China by [33] who advocate for community-based bottom-up initiatives to formalize informal governance. The findings reveal that governance arrangements revolving around food vending practices in Ibadan are structured only around food safety, as this is a government priority. However, provisioning diverse food is not adequately taken into consideration in these governance arrangements. Further research is needed to understand what aspects of governance arrangements can shape food diversity in provisioning practices among the food vendors and improve nutritional health and food security of out-of-home food consumers.

The study revealed that the effectiveness and implementation of the rules and regulations by food vendors was due to the involvement of the food vending associations. These associations play an intermediary role in terms of collecting levies, monitoring, and supervision. This shows that associations are the interface between the government and food vendors to bring about effective and quality outcomes in food vending practices. Governance can be more effective and sustained if the intermediaries are from the sector which the governance arrangements are intended to govern. It is sometimes stress-free to implement and execute rules and regulations because associations are involved in the arrangement in terms of implementation and execution. This shows that informal intermediaries are crucial when formal governance interrelates with the informal structure. This can further relate to the possibility of taking the advantage of informal intermediaries to improve the food diversity provisioned if it becomes a priority. Furthermore, the government and food vending associations can use the advantage of co-governance, informal intermediaries, and periodic training to influence the food diversity in food vending.

Periodical training has been instrumental to the changes in food vending practices when tailored towards health and safety practices. This has been an important initiative that has shaped food vending practices in Ibadan. [61] proper training about safe food handling may be helpful to overcome the challenges faced by food vendors to maintain the hygiene of the foods sold. Food vending associations are the platform that brings all the food vendors together and serves as an important informal instrument for controlling and monitoring food vending practices. It checks their practices in every area and because of the activities of the food vending associations, individual food vendors are more conscious of the kind of activities they take up in their food vending outlet.

There are self-directed practices of food vending that further strengthen hygiene practices and attract consumers. The findings indicate that individual food vendors have additional self-directed rules that further guide their practices and it is within their discretion that they implement these rules. These practice elements mainly concern the type of foods provisioned as vendors have agency on whether and how to construct their menu. Building on the self-directed initiative provides an opportunity for intervening in the food diversity provisioned. This implies that food vendors may influence the diversity of foods consumed by out-of-home consumers if it is considered a priority. This aligns with the findings of [7] that the decision on the number of food groups provisioned at the food vending outlet are at the discretion of individual food vendors. Likewise, more vegetables and fruits can be provisioned to increase healthy food consumption, i.e., more vegetable soups, and menus can be included in their menu settings. Some food vendors reported that these discretional rules are a must-follow for them and their staff as they understand the importance of their services to society. These rules are made and implemented for more effective out-of-home food provisioning practices.

In conclusion, the study highlights governance arrangements that revolve around informal food vending activities in urban Ibadan, how they are being up taken, and the synergies between formal and informal stakeholders in the governance arrangements that concern informal food vending. The study revealed that there are synergies between the food vending associations and the government in the governing of informal food vending whereby the food vending associations play a crucial role. Over time this governance approach has kept informal ready-to-eat food vending practices on the right track. The study also identified the importance of periodic training and the role of informal intermediaries in the governance structure of food vending practices as they aid improvement in terms of functioning, operations, and performances. Consequently, the study revealed that there is also a form of co-governance that involves the government and food vending associations in making and implementing rules and regulations that guide informal food vending operations. The policy implication of the study is that policymakers, government, and food system practitioners should take (formal-informal) co-governance and informal middlemen into consideration in the policy enactment, execution, and implementation concerning the informal urban food supply system. The interconnections between governance practices and the capabilities that this may influence going forward are yet to be researched, for which the findings of this study provide a starting point as it offers suggestions for further research into the food vending capabilities that may be influence by governance arrangements.

## Supporting information

S1 File Interview guide and data for the publication. (PDF)
