# Supplementary material for: Informal ready-to-eat food vending governance in urban Nigeria: Formal and informal lenses guiding the practice
Source: PLoS One. 2023 Jul 13;18(7):e0288499. doi: 10.1371/journal.pone.0288499 (PMC10343139; doi:10.1371/journal.pone.0288499)
Supplement: S1 File — (PDF) [file pone.0288499.s001.pdf]

Interview guide and data for the publication

1. Do the government officials check on you based on different types of foods you provide, if yes, what do they say about the kinds of food you provisioned? Is there any official rules as regards the types of foods provisioned or what really do they base their visit on?
2. Do the officials instruct you or advise you on the type of foods to provision? Have you received any education on food nutrition and healthy provision, if yes, how have you applied them?
3. Can you tell us the government rules that guide food vending practices? Is this rules written on paper and given to you or how do you know about them? Are there any existing and new rules guiding the practice?
4. Do you pay levy or tax directly to the government? If yes, how do you pay it, into the government account by yourself? Do you pay per month or annually? What is the average amount you pay?
5. What is the nature of fines given to you if your default any of the government rules?
6. Do the government official provide training as regards your practice? If yes, in what areas of the food vending practices do they train you?
7. Do the government officials visit regularly? Can you tell us how your relationship has been with the government officials? Is it friendly or with hitches? If hitches, what causes it?
8. Are there template given to you by the government officials as regards the type or number foods to be provisioned?
9. Can you describe the level of participation of food vendor in the policy or regulations that affect them?
10. From your own perspective, In ways can government make regulation or what regulations can they make that can help improve food vending practices?
11. Is there any food vending association in your area? If yes, do you belong to the association? What is the role of the association as regards the types or kinds of food provisioned? do you register with the association and do you also pay levy to them? Do you attend their meetings and is the meeting on weekly or monthly basis?

12. Can you tell us some of the rules of the association regarding food provisioned? how does the rules apply to you? Do the association considers food nutrition and security as priority as part of their association goals? Do you receive food nutrition education from the association? if yes, how do you apply them in your practice?
13. How do the association coordinate the activities of their members? Do they have rules on the kind of foods to provisioned and how they go about it for people to obey the rules? Who and who enforce the rules and how do they go about enforcing it?
14. Can you tell us how your association operate? (asking the association leader). How does the association rules align with government rules on food vending practices? is there any collaborations between government and food vending association? If yes, how does the collaboration play out?
15. Is there any society norms apart from government or association rules guiding food vending practices in your area? if yes can you explain what it is and how this is being implemented?
16. What is the influence of community norms on the provision of diverse foods by food vendors?
17. Apart from government and food vending association, are there any private organizations showing interest in the food vending practice? If yes, can you tell us the aspect of food vending that show interest them? What is the nature of their interest?
18. Are there private organization working with food vendors, what do they bring to the table for food vending in terms on guiding, advising, and exposure to new practices
19. Can you tell us the challenges you face from government officials and how does their rules impede or promote your practices?
20. Can you tell us the challenges you face from association leaders and how does their rules impede or promote your practices?

1. As government agent who supervises food vending activities officially, what is your modi operandi? 1a what are elements of governance you apply in your operation and how do you apply them?
2. Do you organize training to food vendors? If yes how do you go about it? 2a. Can you explain the modality of the training in terms of content?
3. Do you have a stipulate rules that guide food vending activities if yes, how do you apply them? Are these rules written down and given to the food vendors? What are these rules?
4. What are the penalty for the defaulters and how do you implement this?
5. What is your relationship with food vendors and food vending association leaders look like? Can you tell us in details about this relationship?
6. Can you tell us the different aspects of food vending activities you oversea and how do you do it?
7. Do you have rules and regulations specifically on the type of food to be provisioned, if yes, can elaborate more on this. Is their a template as regards this? 7a. In terms of supervision, what areas do you cover?
8. Do the food vendor pay fees or levy to government? If yes can you explain the payment channel?
9. In your own perspective as government agent, what do think you can do more to increase or improve the practice street foods provisioning to a standard?
10. Can you tell us the challenges you are face supervising food vending activities and how does it impede your activities?
11. Do you have any collaborating arrangements with food vending association leaders, if yes, can you explain those collaborations?

Theme	Traditional	Processed	unprocessed
Coordination (govt)	Both of the arm of government officials use to visit me (i.e Public Health officials or Environmental Sanitarian). Yes they use to visit regularly, especially every Thursdays of the week. Government officials from both state and local government use to come. We have different type of them that use to come like sanitarian, tax officials and	It is only Local government officials that use to visit me. (i.e. Public Health officials and Tax collector	

	others that I don't know how to call their names. Mostly visit from government officials.		
Role of visiting officials	Whenever they come, they use to check the environment whether it is neat or dirty and my environment is always neat because I am using gas cooker. The health officials always warned us against the danger of environmental pollution, especially in the community where the food vending business is located. Unpleasant odour emitted by the wastewater and other forms of refuse are inimical to the health of the members of the community. Yes, they give official rules about how decently dressed the food vending staff are expected to appear at work. We must not keep long nails, we must cover our hair and mouth when cooking and dishing out food for our customers. All there rule are expected to be mandatory but they hardly enforce it. The government officials from local and state governments, when they come, ensure proper inspection and registration of all food vendors in the area through the food vendors association. They supervise food premises (Environmental Health officials), all the canteen staff involved in food preparation and equipment used for food processing and preparing the food (by food hygiene and safety officials). They also ensure adequate waste disposal as well as vermin and fly control in the canteen and educate us on good food hygiene and safety practices such as: covering head, cut nails and general soundness of health those involved in food preparation and presentation.	Some of the rules by the government that guide food vending practices here are basically provisioning of healthy foods, personal hygiene of all members of staff and general cleanliness of canteen environment. they use to stay till we completely clean our area by sweeping it properly. And during this time, nobody is permitted to sell anything until we are done with the cleaning. There are three categories of government officials that come here regularly.<br>A. The Environmental Health officials<br>B. Food hygiene and Safety officials<br>C. Internally Generated Revenue officials	Both Local and State government officials use to visit me (i.e Public Health officials or Environmental Sanitarian, Tax collector).<br>The Environmental Health officials come to inspect my canteen and ensure that everywhere is neat, decent and safe for people to eat. Each time they come they usually encourage me to keep on maintaining hygienic environment and if they notice any area that needs to be cleaned, they will point my attention to it and make sure I do it before they leave.<br>Food hygiene officials check the food I prepare for sales on the face value. They come once or twice in a year. They also encouraged to cover my head, take care of my nails and appears generally neat before I start to sell food for the customers.<br>The last group of people come and ask us to pay certain amount of money to the government. Although, these ones are no more coming to collect money as we are now paying through our association to the government. I pay #100 to the local government and #200 to the state government every month.
Environmental cleaning	Whenever I get to my shop every day, I will pray and tidy up the environment and they have known me for that. The government officials, especially the environmental officials visit my canteen every Thursdays to check the level of compliance to government rules on environmental sanitation through out all the market areas in the state. Like me, they just greet me and pass. I even have the phone number of most of them. Because I am the secretary for food vendors' association in this area, the whole of Apata zones (zone 1 & zone 2) besides I am a compliant citizen. I make sure I clean my environment regularly.		
Instruction/govt rule	They use to instruct me to make sure my environment is always neat and we ourselves should always dress neat and be clean. The government rules guiding food vending practices is that the environment must be always neat, they must not live their head uncovered, they must not keep long nails. There is rules given by the government that guide food vending practices is the neatness of the environment and the vendors	The government rules that guide food vending business are basically on personal and environmental hygiene. They are like written constitution, well documented in a single book and I get this book from the government through the food vending	That time they used to give us laws like sweeping, making our stuffs neat and presentable. They also used to talk about whether we stay close to the road or not. Aside from that, they don't say anything about what we sell. I ensure that I keep to the general environmental and personal hygiene as advised by the government officials. I sell fruits

	themselves. Yes, there are existing rules guiding the practice of vending and for now, there is no new rule guiding the practice. All these rules are written in a document given to every registered members of food vendors association. The existing rules are still valid while government officials joined forces together with the ministry of health to educate us on ideal mode of sitting arrangement and provision of other hygiene facilities to prevent the spread of Covid '19 virus among those who come to eat from our canteen. Whatever we want to sell in the morning must not be cooked the previous day. For instance, beans must not be cooked at night and been sold in the next morning, it must be cooked fresh. The government rules that guide food vending practices are about the quality of food prepared, health status of the food vendors to be checked in a government accredited hospitals and proper waste disposal to ensure safety of the people living in the community where the canteen is located. Yes, all these rules are well documented. The government officials issue some of these documents to us when they come so that we will not claim to be ignorance of them. The new rules from the government are about keeping to the Covid '19 protocols.	association. Yes, there are existing rules on the dressing and other physical appearance of the food vending staff, how healthy they must look or appear and avoid involving sickly staff members in the daily activities of the business until they are sound again. There are also new rules as we are already in the 'New Normal Era' due to Covid '19 pandemic. Our canteen spaces must not be overcrowded, washing of hands with soap under running water and checking customers' temperature with infrared thermometer. The rules the government give us is we should not leave our food uncovered. For instance, this puf puf must not remain inside the sieve, it must be put where the flies will not have access to it. Also, we that sell food must be always neat in the way we make it and also dress well. Everything we are using must be handled properly and keep neat.	and there are rules that I am already aware of as a member of fruits fruits sellers association. We have been inform by the government officials not to sell unriped fruits or spoilt ones. So, each time they come like that they will check the environment first and inspect my fruits as well since it is not an hidden commodities. Yes, but in the area of not using harmful substances to preserve fruits or to force the unripe fruits to ripe for immediate sales. They encouraged us to wait patiently and adopt traditional methods which I am already aware of right from when I was learning it from my parents. You know some people make use of spoiled pepper, tomatoes and onions that already have maggots to prepare soup for their customers. This is one of the things they discourage us from doing. I try as much as possible to use all the lessons I learn from the program in my food vending practice.
New rules	More so, there are no new rules due to the emergence of Covid '19. Now, use of facemask is compulsory. We must also ensure social distancing among those who come to eat.	Use of face mask is compulsory for every one including those of use that deals in fruits selling either at wholesale or retail levels.	
Not written on paper	No, the rules were not written on paper, I was only been told by the officials. There is no new rules guiding the practice of vending. No, the rules are not written on paper, we were only been told by the officials	No, the rules were not written on paper, they only use to tell us.	
Influence/application	That is the reason while and tying scarf on my head because it is not good to open up your head while selling food or have long nails on. I have been able to apply them in a way that I am always neat and always cover my head and likewise in the way I made my snacks to sales.		
Vendor policy participation	I cannot answer that whether vendors participate in the policy or regulation that affects them. They use to make regulation that they will later not carry out.	I can say the level of participation of food vendor in the policy or regulations that affect us is high. However, we don't participate directly. The government	Yes, we are being carried along but like I said earlier, they do this through our association leaders who represent and defend our interest and come back to give us feedback at the association

	I will just say that the government should give them the amount of money they can afford, this will make them happy. No, there is no private organization working with food vendors. No, it is the leaders that use to make the policy decision. No, they are not been carried along. Yes, we are included in the policy decision and implementation just that only our association executives are invited for this purpose. Anytime the government wants to make a new rule like the issue of food cupboard that I told you of, they will gather all the food vendor association leaders together and discuss the new policy with them. So whatever they discuss and conclude will then be made known during the association weekly meeting. Our leaders will inform us about the new do's and don't's.	officials usually invite our executive members for discussion and opinions on matters that border on Customers welfare and food vending best practices.	meetings level. Our leaders are being carried along on this. They only come back to us at the association meetings to inform us about new policy. Yes, we are being carried along as we are allowed to deliberate on some certain issues that affect us. Except at the leadership levels, foods vendors are not seriously involved in policy making as such. Our leaders are usually invited when government needs information on new policy on amendment of old policy made by government on food vending practices.
Food type rule	No, they didn't check on me only based on the food I provisioned. No, they did not use to give me instruction on the type or number of foods to be provisioned, because, they cannot teach me my job. No, there is no template as regards the type or number of food to provisioned. It is only those that sells amala and rice that they can give such thing. I am the one that knows how I bought the flour I use and somebody will now be given me template for it. They didn't check on me because of the food I provisioned. No, they did not give instruction on the type or number of foods to be provisioned. They did not give me any template as regards the type or number of food to provisioned. they train us based on the type of food we have chosen to provide for the public. We have attended series of training on food nutrition and healthy provisions. I can't say pointedly that the association regulate what we sell. Some people sell varieties of food depending on their capability. Yes, the association considered food nutrition and security as we are regularly being educated on this. The rule about numbers of food foods or type are based on external factors such as customers patronage and availability of raw food materials in the market.	The government officials do not instruct or advise me on the type of foods to provision. However, they organisation periodic seminars and workshops on food nutrition and healthy provision. There is nothing of such. We prepare the type and number of foods we chose to prepare and provisioned. Yes, they consider food nutrition and security as priority as part of their goals.<br>Yes and that is why I sell my food with different type of soup and meats.	In addition, everyone will sell what she knows is needed by those living in that particular area. This area is dominated by local people. They are not the type that want all these light foods, what they want is heavy food that can sustain them for a long time. Assuming it's area like Challenge here in Ibadan people can still be demanding for jollof rice and the likes.
Education on food provision	No, I did not have any education on food nutrition and healthy provision. The government officials have educated me on food nutrition, and healthy provisions. I am applying them by keeping my environment clean, covering my heads and ensure my foods are well covered to prevent contamination by the flying flies.	Yes, I have received education on food nutrition and healthy provision. I have my OND in catering and I have the certificate. Yes, we normally have seminars and workshops on food nutrition and security.	
Tax/levy	Yes, I pay tax directly to the government. I use to pay it yearly and it depends on the amount they bring and this is always being determine by the government in power. Like the one I paid last is #3,500 so, it is possible for it to increase or reduce next time because they have not bring	No, we did not use to pay tax directly to the government. It is our association leader that use to tax on behave of all the members and this is been contributed	I pay tax into government accounts using the account details provided by the government officials. Other monthly are paid indirectly to the government through the food vendors association.

	the amount for this year. Yes, I pay it directly to the government. There is one for every month and another one for every year. So, I used to pay monthly and yearly which is either #300 or #700 before to different government officials whenever they come around and it depends on the amount they bring that I use to pay for tax or levy. I pay levies to both state and local on monthly basis through our association to the government at each levels. Before food vendors association was saddled with this responsibility of collect money on behalf of government officials, they used to come directly to approach us to pay local and state governments levies but I think due to clashes between food vendors and some of these impatient government officials it was later decided that the association should take up this responsibility. I pay #200 to the state government purse and #100 to the local government purse both on monthly basis.	zone by zone by given ticket to the members and later pay it to the state. Yes, I pay levies through the food vending association to the local and state governments. No, I don't. I pay #200 monthly levy to the state government and #100 monthly to the local government. I made this payment no longer directly to the government officials but through our food vendors association.	I usually get tickets for some of these payment made by me. I pay tax annually and other levies monthly. Some of levie I pay monthly are #100 to the local government and #200 to the state government. These levies are tagged "Daily Hawkers Permit" collected through the food vendors association to the government both at the local and state levels.
Defauting	Whenever I default their rules, they use to carry my foods to the secretariat and fine me for rule defaulting in which I will later pay for the fine. The nature of fines given is by tying rope round the person's shop and we will not be able to sell anything until we go to the secretariat to pay the money we have been fine with.<br>If the environment is dirty, they will lock our shop and they will ask us to go and pay to Eco bank. Like some time ago they lock my neighbor's shop because she put small dirt inside sachet water nylon pack in front of her shop and they ask her to pay #1000. The small grasses you see there, if I we did not remove it before Thursday they will fine us by locking our shop.	They will lock the shop of the person that defaults their rules and ask the person to come and meet them in the secretariat. If it is during WHY that the person cook. they will carry the person's food to the secretariat where she will come and bail it. There is no specific rule on the kind of food I provision but there are task force members of the association that go about monitoring the level of compliance of the association members when there is a general rule that every member must comply with. The association leaders enforce the rule and sanctions erring members.	Anybody caught disobeying any of the hygiene rules especially the ones put in place by the government, such person will be severely dealt with. The person's shop will be locked up and the person fined. Until the person goes to their office at the local government, Ido, the person cannot sell food. At the local government that is where the defaulter will be told how to pay his/her fine. They might tell the person to pay the fine into a certain bank account but I don't think they collect money from anyone directly.
Formal and informal Traning	Yes, the environmental sanitation use to do training for us. The training is always once in a while and is still about the cleanliness of the environment, how to dress while practicing food vending, how to wash hand and cover our body. Yes, they use to train us. We use to go to Onireke at the local government secretariat for the training and we used to pay for the training. They trained us on how to prepare our stew or soup without too much of maggi, how to relate with our customers and also how to take good care of our environment. Yes, they used to provide training as regards our practice. They trained us in all areas of food vending and they also award certificate to those that have been in the food vending practices for long.	I do apply them not just to avoid being sanctioned but to attract customers, for they will only patronise food vendor whose environment is appealing and provide tasty foods. The training is once in a year and we used to pay #2,500 for the training. They use to teach us how to prepare vegetables, ewedu, rice, beans, they use to bring caterer for the training. they will first come and collect the money and later tell us when to come for the training. After which they will give us injection and after that trained us. Big	Government officials provide training for food vendors occasionally. Most of the times these training are organised, they are basically on personal and environmental hygiene. Also, they train us on customers and food vendor relation, how we are to prioritise and mind first the health of our customers above our monetary gains. Payments of levies by the members of the association is their priority not nutritional education or security.<br>All they are concerned about is the quality of food being offered for sale to the public. Yes, government organises training for the food

	Occasionally, they organise programs on healthy food awareness for the food vendors. Of course, they will ask us to pay a token and they usually give us certificate of participation.<br>I usually apply some of the training they give us on the importance of keeping our food warm and make our environment customers friendly. Of course, we get invitation from government officials to attend seminar or workshop where they talk about food nutrition and healthy provision. Not as if they are trying to teach us how to go about our occupation that we have been practising for years. Although, government irregularly organise workshop for us through the association and most times our discussion is mainly of environmental hygiene and personal cleanliness. Yes, they provide training as regards the food I provisioned.<br>They use to bring people to train us on different types of food. We used to pay money for the training but the amount depends on the type of people that comes. Also, we use to do test in the Local Government hospital once in a year. They can ask us to bring sputum, stool or urine samples. We use to pay #2,500 for the test. After the training, they use to give us certificate.	restaurant like Iya dunni organises training programs for smaller food canteen. It serves as an avenue to encourage and motivate us to do more and aim for the best in life.<br>The government officials provide training on the area of provisioning of quality food and maintenance of personal and environmental hygiene.	vendors on food nutrition and healthy provisions at least once in a year. Yes, at least once a year, the association organise workshop and seminars basically on personal and environmental hygiene. Perhaps, they believed we are professional with adequate knowledge on food nutrition as it determines the level of patronage we get at our canteen location. Well, since the focus as always been on hygiene, I make sure I enforce this among my staff. Also, I employ the service of private waste management company who comes regularly to pack the waste at my canteen.
Relationshipt with govt official	My relationship with the government officials has being friendly. We did not use to fight because I used to obey their rules. Can you see my hands, head and environment? I did not keep long nails, my head is covered and my environment is also neat. I did not use to fight with them whenever to come. Therefore, I have friendly relationship with them	They can make regulation in collaboration with our leaders (Iyaloja, Babaloja and Baale) for them to be able to get to us and vice versa	
Association r/ship with govt	There is cordial relationship between the government and our food vendors association as they help the government to monitor level of compliance to government imposed rules on the food vending practices and collect some levies on behalf of the government and remit to government account. The government go through the association to get to know relevant policy to make concerning our business and also communicate through the association to us whenever they want to organise seminar or workshop to enhance our business and ensure the safety of our community. Association rules apart from the ones that borders on members punctuality in the meetings and same as governments rules. Our association have good relationship with the government as government relays information to us through our association leaders.	Yes, there is working relationship between our association and the government. Governments see the association as a platform to gather the food vendors and address us, train us and announced the policy of the state that has to do with our business.	
Meetings with governemnt agencies	The only way the association use to operate is whenever there will be meetings with the government officials, they use to make circular to the	We participate in policy making, especially the one that affect us as food	

	leaders in the association and the leader will inform the members in her areas. If the leader is the only one that attended the meeting, she will come and give feedback to the members when she comes back. The association use to sell Ankara to the members for their anniversary at the rate of #2000 and everyone must pay the money.	vendors but indirectly, i.e. through our association leaders.	
Benefit from govt	Also, those that use to hawk in the sun, if they give them space to stay to be selling their foods. They can as well empower them by given them equipment that will enhance their food vending practices. There are many ways the government can make regulation for instance now that food stuffs are too costly, our government should help us find something to do about it. Because whatever you buy from the market, that is what you are going to sell. Like this cassava flour that is #5000 before is now #30,000. And this is because people are running away from the farm because of insecurity nobody want to go into farming again and that is the reason we have limited farm products. Likewise, the government did not allow the importation of rice into the country and this has make it to be too costly. Government can help us reduce the amount of money we pay both monthly and yearly and make us feel the impact of the money we pay as I can barely acknowledge the direct benefits of some of these payment we made to the government. The regulation they can make is not more than that they should not make things too costly. Because it is the things that are costly that affected some of us and this make them to leave food vending practices, because they cannot manage it. If things are not costly, sales will be encouraging. Because we have to reduce the size of some of the things we sell and this have make some customers to stop patronizing us. Like the bike men, that use to buy egg roll and drink coke with it and be satisfied has stopped buying since it has depreciated in size due to it. They should help us influence government decision to support us with funds that will make the growth of our business visible and beneficial to the populace. They should make rule that all members must be duly registered to have our data in case government finally decides to assist us with funds.	In my own perspective, the government can make regulation in a way that it can improve food vending practices and this can be done by providing conducive place for those practicing food vending in an inconducive place.<br>It can be done by building shops for the vendors in the areas their food vending practices can be improve. Likewise, by reducing the prices of the ingredient used will go a long way in improving food vending practices. For example, bean cake was sold #30 per one before increase in the amount of beans, pepper, vegetable oil and the rest and it is now sold for #50 per one. The government can help us to talk to all the people in charge of all these things in order for them to reduce their prices. You see, oranges, cucumbers and pineapples are small business for people who don't have sufficient funds to run a big business.	In my own opinion, government can make regulations in the areas that border on economic and finance, as these areas have direct impact on our business. The government can help us by regulating the hikes in prices of food raw materials we buy to make ready to eat food available for our customers. Some of them are complaining about the quantity of food we sell and at the rate we sell. This is obviously not out faulty but general inflation in the prices of commodities in the market. Also, if possible, let government subsidise the prices of food raw materials by helping the farmers who are our main suppliers of main food raw materials. What I think the government should do is that, they should organize empowerment program, so that we can have the privilege to train who are less privilege people and through that we too will benefit from it. Because by the time they pay us, we will be able to use it to support our business. Many people are saying that they cannot learn food vending practices because of lack of money they will use to enroll themselves for the training. So, if the government organizes that, it will reduce the money they will pay for the training and by so doing food vending practices will improve.
Visitation frequency	Yes, the government officials use to visit us regularly. Government officials do not have specific time they visit us. They come at will and because I know they can appear at any moment in the day, I ensure they have nothing to say against my canteen by keeping to the regular routine	Yes, they use to visit every Thursdays of the week	

	rules that guide food vending business. I have always maintained friendly relationship with the government officials because I know what they want in terms of keeping to the rules.		
**Informal**			
Association/ hierarch	Yes, we have association in our area and I am a registered member. The role of the association is to ensure that no two members should sell the same type of food in the same place. Yes, am a registered member, I have my certificate and I always pay my levy to the association. Yes, I do attend meetings every Thursdays of the week. The association comprises of different types of food vendors and always see to the affairs of the members.<br>They collaborate with the government. Our food vendors' association consists of people selling traditional foods like amala, rice, beans and so on as well as those selling snacks like puff-puff, buns and so on. Those selling fruits have a different assistant where the attend. They are the ones who pay the government whatever they want to pay on behalf of all other food vendors. I am even an executive member. The association ensures unity among members. To start food vending practice in this area there are a number of steps to be taken. The association will take the person through (paid) trainings and seminars to ensure the person becomes familiar with the practice. After which a certificate will be issued. The person will register with the association by paying a sum of #10,000, 2 cartons of biscuits and a crate of soft drinks. The person will also be given the association card and printed rules. The meeting holds every Thursday by 4pm.<br>We pay #200 weekly to the association. This money is used to buy clothes for our social functions. Also if anyone needs assistance, we give the person from the money contributed. Yes there is and I belong to the association. It is even a must for everyone selling traditional food to belong if such person doesn't want trouble from the association.<br>The association has a five member executives in charge of the association. The president, vice president, the treasurer, the secretary and one other person like that.	Yes, I am a bonafide member of the food vendors association in my area. They ask us and registered the type of food we provision. Their main role is to ensure that no two food vendors selling the same type of food stay together at same place to avoid clashes. I must say that that rule was before, now five food vendors can stay together at one place, without any form of clash at all. We all know our customers and our customers know us by the taste of of our foods. I registered with the association and pay sum of #5,000.00 as registration fee. I attend meetings on every Thursdays and we contribute to the association in every weekly meetings.	There is no association for those of us selling snacks in this area. Therefore, there is no association rules guiding us and no one is monitoring us. There is no body coordinating our activities. No, there is no community or societal norms. I heard that once one is a registered member of the association, one is free to sell all kinds of fruits. Therefore, I don't think there is any definite rule determining or limiting the type of fruits one mist sell. The association has a 5 members executives that coordinate the association as a whole. They include the president, the vice-president, the general Secretary, the treasurer and the taskforce leader. I cannot tell because I did not use to pay fines. I always try my best to obey their rules and by so doing, they did not use to fine me. We participate in policy making, especially the one that affect us as food vendors but indirectly, i.e. through our association leaders. Yes, there is food vendors association in this area and I am a registered member of the association. The association requests that I should register the type of food I want to be selling and I did so. I registered with #10,000.00 and give one crate of soft drinks and carton of biscuits. I was requested to go for medical check up to determine the level of my fitness for the food vending business. I normally attend the meeting every week and pay #200.00. If I cannot go on a particular meeting day, then I have to send money for the weekly meeting through someone.
Association rule/role	Members selling food must be separated from one another at least with six pole away from one another.<br>Those that refuse to join the association and are practicing food vending will not be allowed. Their goods will be collected from them. All members must fully participate in all the association meetings. No member must come late or absent from the meeting without prior notice. We must not snatch one another employee.	Yes, they consider food nutrition and security but I believe they give more priority to payment of Levy more than anything else as their interests is basically on monetary gains. One popular rule of the association I can remember now is that if one person has been selling food in a	Some of the rules of the association are derived from the general rules enacted by the government. They are basically on personal and environmental hygiene. Yes, the association monitor level of compliance to the rules guideng our business by the association members through the assigned task forces who are also members of association in the

	Anyone that default the rules will be fine. Their rules only align with the government in the aspect of neatness. Our association rules are the same with government rules, in fact I can say that our association is just meant to implement those government rules on food vending practices. The association is the channel through which the government communicate us on general issues, so I can say government collaborate well with food vending association. Also, we pay monthly to both local and state governments via association. The association executives now in turn remit the money to individual government account. The leaders of the association have our data, therefore, to coordinate us is not a challenge. They have their way of detecting those who have not registered as members of the food vendors association.<br>There are 21 hygiene rules guiding our practice as well. They printed on paper and approved by the government. I'll mention few of them; the head must be covered, the food vendor must not sell food if she has cold/ running nose/ cough, one must not talk while serving food so as not to spit into the food, we must not pick our teeth while serving food etc. As I am talking to you now, if I want to attend to customer, I'll have to stop talking.	particular location, if another is going to be selling the same types of food, the association will instruct the person to count seven electronic Poles distance away from the the one who has been there before the new person came. The association rules are by products of government rules. Yes, there is. The government interact with the association leaders and they are tools in the hands of government both at the state or local government.<br>One, two vendors must not have their outlets too close to each other.<br>Two, if they are inevitably close, then the association will say that one of them should sell swallow type of food while the other person can sell food items eaten with spoon e.g rice, beans etc.<br>Three, if a vendor has issue with her outlet location maybe it was demolished or maybe she was sent away and she now gets a new location close to someone else's outlet then the two of them can continue selling what they sell even if it is the same type of food.<br>Four, if someone new comes to situate her outlet close to mine, I must not take law into my hands. I have to inform the association, they know how to settle it amicably.<br>These are few of the rules and they to ensure unity among us.<br>It is the government that organises food nutrition education for us not the association.	same occupation. Another thing is that if you want to start selling food anywhere around here, you must be at least 10 electric poles far away from the nearest food vendor to you. All these are because we want peace to reign in our midst as association members. Before now there used to be different types of conflict to the point that we didn't trust one another. When a food vendor visits another food vendor, we'll be looking around to see if the other person did not come around to pour some kind of charm on the floor. So for all these to stop, we had to put certain rules in place. Nowadays, we eat together and attention social functions together without having any negative feelings towards one another.<br>The programs we hold are seminars and trainings for new members. Aside that we do have annual party.
Association functions	They coordinate the activities of their members by ensuring there is no clash between any members based on what they provisioned. Basically, the role association plays is just to ensure peaceful coexistence among food vendors in the same locality and monitor level of compliance to the rules guiding food vending practices in my area through the association task force. However, our association sometimes inform us and prepare	The association coordinate the activities of the members through regular weekly meetings where matters that affect food vending practices are discussed. Except in the situation where there is clash among members of the association on types of	Our association emphasised cleanliness, regular payment of dues and punctuality at the weekly meetings. They also emphasised that those Hawking food should stay far from those sited in a particular location except if they are not selling the same food items. Actually, this rule are no longer

	us to be addressed by some food related none governmental organisation. There are mostly on nutrition, providing healthy food for the public. If there is any important rule they make, there are taskforce who are responsible for monitoring compliance among the members and report any defaulter.<br>I can say government rule is the same as association rules as because the formation of the association is with the consent of the state government. Yes, the association collection money from us and pay the government. From time to time our association leaders rub shoulders with the state government. The state government give instruction that they enforce on us as members of the association.<br>We emphasised on not using harmful chemical substance to force unriped fruits to get ripe fast. We also emphasised on personal and environmental hygiene. Sometimes the association raid to check the level of compliance among members who sell in that neighbourhood. We share helpful information among ourselves when we meet in the weekly association meetings. I apply them by making sure that I want my fruits well before offering them for sales. One of my customers has been motivated to buy fruits from the reason of the level of hygiene I maintain at my selling points.	food provisioned, the association hardly talk about types of food or impose any arbitrary rules on type of foods to be provisioned. In case of clash, they usually device a means to settle it amicably among members of the association and send representatives who will come and inspect the level of compliance and feed the association back on the matter. In case of any forms of insubordination, the association may decide to impose fine on the defaulters. The association coordinate our activities by given us some instructions at our weekly meetings. Our association, when it was functional, has no rule that guide the types of fruits we offer for sales. The association leaders enforces any compliance to the known rules.	an issue as individual food vendors knows how to attract and maintain our customers. There are association task force who patrol areas where an issue about food provisioning leads to clash between two members of the association. The usually go round to check level of compliance among the members concern and if it on general rules, the task force of the association will still check round to ensure defaults are called to order..<br>Yes, sometimes the association will invite experts of food nutrition to talk to us. It actually does not affect number of food provision since individual customers will have their choice made at our canteen based on the available food items. However, I keep to those rules that has to do with hygiene.
Association meetings	Yes, I do attend meetings every Mondays of the week. . The payment of the levy as the newly admitted member of the association can either be paid once or on instalmental basis as it is convenient for the new member. The meeting usually holds on weekly basis and I attend meetings regularly.	The basic rule of the association is that you must not missed meeting and your must make sure you are paying your weekly dues. Yes, if one is absent for about three weeks for none genuine reason from the meeting, the person will pay fine of #500 compulsory.	
Association coordination	They coordinate the association by calling us together for weekly meetings where we deliberate on the matters that affect us. Actually, the focus of our meetings times is on how the leaders of the association will "milk us" for money again. We are not really enjoying the direct benefits of this money we are contributing every week. I am simply tired of everything but the association is compulsory else, they will start to come and disturb me for not cooperating with them.	The association executives use to come and check on us to know whether we keep to the rules or not.	The association has a 7 members executive panel that controls what goes on in the group. This includes; the president, vice president, general Secretary, treasurer, assistant treasurer, youth representative and the association kind of police. They are all females.
Personal development	This can happen through personal development in one's area of interest in food vending business. This I think will improve food diversity provisioned.		
Personal rules	Yes, I have my own rules that guides me in the type or number of food I provisioned. My own rule is once a customer buys anything from me and	The personal rule I have made for myself here is about time. I must wake up at 5am	I always make sure that buy what I can afford to sell at the prices my customers will not find

	bring it back, I will not collect it back for the sake of life. Once you buy something from me, is not returnable. Likewise, I have to be careful in the way I fry the plantain chips.	to start the cooking and I closed from here latest 12noon. This is because the food I prepared is mainly for breakfast.	difficult to buy my fruits. As you already know that fruits are highly perishable, one cannot keep them for too long.
Community norms	Well about societal norms guiding food vending practices, not as much as I know. However, the community where I operate my business being a civilised environment specified time at which movement is allowed for security reasons. I usually ensure that I abide by the time stipulated by the community leaders. The norm of the community here is that there should be peaceful coexistence. This is to prevent rivalry or a kind of unhealthy competition among the food vendors in the community.	Yes, there is community norms. The norms is that we must not stay here till late in the night. They only allow us to stay from 6am to 8pm.	
Collaboration/private	There is no private organization working with food vendors. Everybody is on his or her own. Is no more like in the olden days that some companies worked with food vendors. Because now, they are after how to make sales. Yes, there is other private organization like LAPO (Microfinance bank).<br>They are interested in all food vending practices by lending them money to support their businesses. There is collaboration between them and the government, in the sense that they used to reach out to the government on behave of the members and vice versa. Those private organisations I can remember very well are those ones who come through our association to train us on new practices that we may either adopt or drop. We choose whether we want to follow their teaching or not.<br>Yes, there are private organisations working with us as food vendors. They educate us on new discoveries and guides us on best practices.	The private organisation that have been here are company such as Coca-Cola bottling company and others. Their interest is basically on the patronage they expect from me buying their products to complement my foods. The nature of their interest is basically on business matters. They are simply after their own profits. Yes, there are private organisations who are interested in what we do as food vendors. Some of them nutritional supplements of the food we provision for the people and encourage us to put into consideration the health safety of the people who rely on us for feeding. The nature of their interests is public safety and welfare oriented.	Well, there may be some organisation like that but not one have been here. Except those that come through our association, organising training for us. Yes, they guide us, advice and introduce new standard best practices but as I said, they do these through our association not commonly on individual food vendors basis. I don't know, except the Microfinance bank that people use to go to and borrow money from to support their businesses. It is only microfinance bank that use to borrow us money that we use to pay back without any problem.
Association support/benefit	The way they can help food vending to improve is by lending money to the members from their weekly contribution. We can help one another by sharing useful information that can help us help our common businesses to grow. Some of us lack surviving or coping strategies when challenges in our business arise but if someone who has successfully scale that hurdles volunteers to help, the person might remains in the business for a long time.	As we normally hold our weekly meetings regularly, individual food vendors with the ideas on how to improve our common business can share to help others in the same occupation. This can be done by sharing our unique experiences with one another on food diversity provisioned and be a source of encouragement to one another. Strive and envy should be totally done away with.	

Support	It is only microfinance bank that use to borrow food vendors of different types of food money to support their businesses in which they later pay back with interest as at due date. The major rules are that we must attend all meetings, ask after one another that means the association encourages all food vendors to be friends. Then another thing is that we must pay our dues at every meeting.	Well, I think we need more orientation to improve on our food vending practices and be updated, properly informed through regular training organised by the association. The association can be more strict in ensuring compliance on the agreed rules by imposing stiff punishment on any default. What give me concern some time is that when we make rules some people will not comply and yet there is no serious punishment to discourage such defiant attitude.	Yes, I am a registered member. The only role they play is to inform us about season of fruits and address one or two challenges any association member is facing. I paid #5,000.00 for registration as member of fruits sellers association. I attend meetings every Thursday at our meeting point down the road there.
Self-imposed	Yes, it is obtainable. Self-decision on the type of food to be provisioned depends on what someone has interest in to be practicing. Self-decision is the decision you make on the type of food vending you want to be practicing. My own self rules that am following is when I employ workers, I used to tell them that they must not leave the plates the customer use to eat without washing it with immediate effect. Also, the quantity of maggi to be use for soup and many others. The rules I made for myself are the ones guiding my staff on the basis of hygiene and punctuality. These rules have nothing to do with the type of food I provisioned. When I started food vending practices, I started by selling only Akara because is the main business in my town and as time goes on I see that it is not sufficient enough and I added moinmoin to it. Still is not sufficient so I have to add other things am selling presently to it. My self-rules is not more than, no customer should stay for long in my shop after eating to the extent of sleeping. I use to tell them to go their house. I make sure I monitor the cooking and personally handle dishing of food to the customers for hygiene reason. This is basically my personal decision.	I made the decision because, many people leave home in the morning without eating breakfast. So, they use to buy bean cake and bread. Especially those people working in building constructing site because it used to sustain them. Likewise, many people eat it as refreshment. As I said earlier the training I had informed the kind of food I provisioned. There is no such rule apart from the fact that I know what I can afford to do. Yes, some customers come to try and influence me to change my food types by adding to it but I tell them these are the food type I am knowledgeable about. Therefore, they should help me eat it with love. They can only suggest, I know my limit. Yes, I have my own rules. Whenever customer buy anything from me, it cannot be return back to me because it is possible they might have touched it and even me selling it did not use to hold it with my bear hand when selling it. Even none of my children can help me sell my snacks anyhow without putting nylon in their hand.	Though some people don't like to eat swallow such as Eba, Fufu, Amala or Semovital in the morning, yet those are what I chose to prepare for those who will prefer to eat such foods. My training was basically on these types of foods. I am interested them and I am not ready to do otherwise so far my customers are still enjoying it and come regularly for it.<br>I have decided to make my customers' welfare a priority. When I sell fruits, only good ones I offer for sale to encourage my customers to keep coming to my place to buy more fruits. I check on them to find out if they enjoyed fruits they bought from me

Challenge	The challenge I used to face from the association leader is the compulsory attendant in the association meeting. Government should please reduced the amount of money they take from us on monthly basis as we have not really benefited as much as what we are being asked to pay.<br>The only challenge that am facing from the government officials is the payment of tax because, how much are we realizing from what we are selling and they will still come to collect money. That is the reason I used to tell my apprentice to tell them that I am not around anytime they come. I am actually not happy with all these weekly payment that there is almost no evidence of the good purposes the association is using the money for. The association leaders should please be accountable to us so as to encourage to continue to give. Then we as members of the association should also have some benefits we can enjoy from what we contribute every week.	The only challenge I used to face from the government officials is when they sudden come and did not allow us to sell anything till all our customers left. So now in order for us to face challenges with them, we use to obey their rules so that we will not be disturb by them.<br>Their rules did not impede but promote my food vending practice. Because if anyone that want to buy something from us sees that our environment is clean, they will be able to buy from us.	I really to have serious issues with the association just that I am tired of their routine. We pay every week, but nothing serious to show for it, whereas their are other food vendors who are not yet members of the association. They don't pay as much as we do and we that paying don't seem to be better off. The association should try to ease the financial burdens on members of the association.

Levy	We pay levies both to the state and local government. Each member from each zone pay three hundred naira monthly to the governments. The money is shared between the state and local governments. The state government takes two hundred naira while the local government take one hundred naira. I, as the association leader will collect this money from all members in my zone, then take it to the local government for onward transfer to the State government account.
Training	Yes, the Environmental Protection Agency or Health officials organise periodic training for us. They trained us basically on hygiene protocols. Yes, I remembered they also trained us on types of ingredients to use for the health benefits of the consumers. Quality ingredients used in appropriate quantity will result in healthy food for public consumption.
Policy participation	Yes, we participate in policy and regulations but at higher leadership level I.e at state level. Although, we sometimes have our input in the discussion, however, at the leadership level, the decision is taken.
Association function	Yes, in fact, I am their leader in this zones. Before now, the food vendors association monitor the types of food each member provisioned to ensure clash free operations among members. If a new member come to register, such will be interviewed on the shop location and the type of food she intends to sell. If no food vendor around the area sell similar food type, then the person will be permitted to operate her food vending business. However,

	in the case of two food vendors selling similar food type, the association leaders will count five electric poles as a measure of appropriate gap between the two food vendors to avoid clash. It used to be seven electric poles distance before, later it reduces to six, now five. Notwithstanding, the counting of electric poles is no longer a barrier as many people are now into the food vending businesses. Our customers know where to go when they want to eat regardless of proximity between two food vendors selling similar food types.
meetings	As association, we registered every bonafide members of the association and we pay two hundred naira weekly on our meeting days which is usually every Fridays afternoon. Regularly, we educate one another in the meeting on the area where members might need to be guided. We share experiences and vital information to help the growth of our business. If any one share information the is new but applicable, I put it to test first before putting it to use. The number of food and type are all my personal decision.
Association/government	Basically, the association ensure that every member comply with the government rules, especially the one that borders on hygiene and any grievance among members should be reported to the leaders for settlement. We have task force that move round to ensure compliance among members. Not often, but the task force move round to ensure compliance and report defaulters to me for appropriate sanctions. Yes, the association monitor the types of food provision to ensure clash free food vending among the food vendors in the same location. If any one is reported defaulting, the association will intervene to settle the clash amicably.
Food provisioning	Yes, we consider food nutrition and security as priority. This is actually one of the reasons for the formation of the association, to ensure that members provide food good for the health of the members of the public.
Association rule	Our association rules are quite similar to that of the government. We are basically implementing government rules. We have strong collaboration with government and have the strong backing of the government behind our operations. We are registered under government, we pay our due regularly to the government, so government cannot deny their awareness of our existence in the society. We execute government decision and implement their rules. Our collaboration with government is very cordial.
Norms	We are to keep to the rules guiding the community where we operate our business. Proper hygiene must be maintained to ensure safety of the community where we operate.
Private	Those private organisations provide guidance advise us on lastest discovery that can aid and boost our business. We ask them necessary questions when they come and get exposed to new practices in our business.

Challenges	The challenges we face most time is that we are not really enjoying much of the benefits of the money we usually contribute and remit to the association purses and local and state governments levels. The association rules are not in any way impede my operation, rather they promote our common business and ensure that we all operate and maintain uniform standard all over Nigeria.
